# A case series of premenstrual disorders presenting to the UK's National Female Hormone Clinic

**DOI:** 10.1192/bjb.2022.63

**Published:** 2023-10

**Authors:** Thomas J. Reilly, Clare-Louise Knox, Michael S. Marsh, Michael C. Craig

**Affiliations:** 1 Department of Psychosis Studies, Institute of Psychiatry, Psychology and Neuroscience, King’s College London, UK; 2 National Female Hormone Clinic, South London and Maudsley NHS Foundation Trust, London, UK; 3 Department of Psychological Medicine, Institute of Psychiatry, Psychology and Neuroscience, King’s College London, UK; 4 Department of Obstetrics and Gynaecology, King’s College Hospital NHS Foundation Trust, London, UK; 5 Department of Forensic & Neurodevelopmental Sciences, Institute of Psychiatry, Psychology and Neuroscience, King’s College London, UK

**Keywords:** Menstrual cycle, menstruation disturbances, premenstrual dysphoric disorder, premenstrual syndrome

## Abstract

**Aims and method:**

We aimed to describe the clinical characteristics of female patients presenting with premenstrual disorders to a tertiary service in the UK. We conducted a retrospective case-note review of referrals to the National Female Hormone Clinic from April 2014 to August 2020. Based on clinical assessment, we determined whether the patient met criteria for premenstrual dysphoric disorder or premenstrual exacerbation of an underlying psychiatric disorder.

**Results:**

Of 146 patients seen in clinic for premenstrual disorders, an ICD-10 psychiatric diagnosis was made in 130 (89.0%); a minority 16 (11.0%) did not have a psychiatric diagnosis. Following assessment, 94 patients (64.4%) met criteria for premenstrual dysphoric disorder and 67 (45.6%) had exacerbation of a psychiatric disorder.

**Clinical implications:**

Patients presenting to this specialist service had complex psychiatric comorbidity; almost half presented with exacerbation of a psychiatric disorder.

Premenstrual disorders are defined by their temporal relationship to the menstrual cycle: symptoms must worsen premenstrually or be completely confined to the premenstrual phase. Examples include premenstrual syndrome, premenstrual dysphoric disorder (PMDD) and premenstrual exacerbation of an underlying disorder.

Premenstrual syndrome affects 20–30% of females,^1^ and is characterised by physical and emotional symptoms during the luteal phase. A minority also meet criteria for PMDD, a more severe disorder with a prevalence of 1–6%.^1^ It is diagnosed by a range of predominantly affective symptoms, accompanied by significant functional impairment. PMDD can be clinically difficult to differentiate from premenstrual exacerbation of an underlying psychiatric disorder, because of overlapping symptomatology.^2^ Irrespective of precise diagnosis, a recent meta-analysis demonstrated worse mental health outcomes, such as suicide and psychiatric admission, around the time of menstruation.^3^

The purpose of this study is to collate consecutive referrals to the National Female Hormone Clinic, based in London, and describe the clinical characteristics of female patients reviewed in clinic.

## Method

We conducted a retrospective case-note review of all referrals to the National Female Hormone Clinic over a 6-year period from the clinic's inception in April 2014 until August 2020.

All patients were seen by a single consultant psychiatrist (M.C.), who has also trained in obstetrics and gynaecology, with further input from a consultant gynaecologist (M.M.). A full assessment for each patient included exploration of the presenting problem (usually related to the effects of female hormones), psychiatric history, gynaecological history and medication history. Treatment recommendations were made based on evidence-based guidelines,^4^ encompassing lifestyle changes, psychological therapy, selective serotonin reuptake inhibitors (SSRIs) and suppression of ovarian hormone fluctuations.

Data were extracted from clinic letters by a study author (T.R.). Variables of interest included: referral reason, age, presenting complaint, psychiatric and gynaecological comorbidity, and psychiatric and gynaecological medication.

Primary and secondary psychiatric diagnoses (according to ICD-10 criteria) were provided as part of the assessment. For the purposes of analysis these were grouped into:

- anxiety disorders (F40 Phobic anxiety disorder, F41 Other anxiety disorders, F42 Obsessive–compulsive disorder, F43 Reaction to severe stress and adjustment disorders, F48 Other neurotic disorders);
- bipolar disorders (F31 Bipolar affective disorder);
- depressive disorders (F32 Depressive episode, F33 Recurrent depressive disorder);
- neurodevelopmental disorders (F84 Pervasive developmental disorders, F90 Hyperkinetic disorders);
- personality disorders (F60 Specific personality disorders, F61 Mixed and other personality disorders, F62 Enduring personality changes, not attributable to brain damage and disease, F68 Other disorders of adult personality);
- schizophrenia spectrum disorders (F20 Schizophrenia, F23 Acute and transient psychotic disorders, F25 Schizoaffective disorder);
- others (F50 Eating disorders, F70–79 Mental retardation).

We recorded whether the patient reported menstrual worsening of depressive symptoms, manic or hypomanic symptoms, psychotic symptoms or migraines. Following assessment, it was determined whether the patient met DSM-5 criteria for PMDD or premenstrual exacerbation of an underlying psychiatric disorder (these categories not being mutually exclusive). As DSM-5 requires prospective recordings of symptoms to make a definitive diagnosis of PMDD, our diagnoses should be classed as provisional. Psychiatric medication at time of assessment was categorised as: antidepressants, antipsychotics, mood stabilisers and others. Gynaecological medication was categorised as: oral contraceptives, intrauterine contraceptives, transdermal oestradiol, gonadotrophin-releasing hormone analogues and hormone replacement therapy.

### Ethics and consent

Clinical audit approval was granted by South London and Maudsley NHS Foundation Trust. As this was a retrospective case-note audit rather than research, patient consent was not obtained.

## Results

**Fig. 1:**
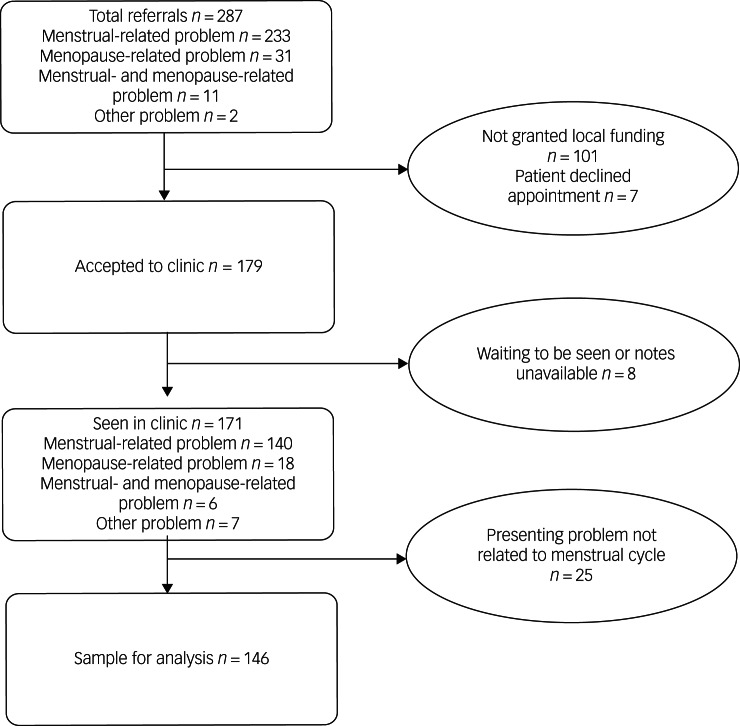
Flowchart of all referrals to the National Female Hormone Clinic from its inception in April 2014 to August 2020.

During the study period, 287 individual patients were referred to the clinic (Fig. 1): 233 for a problem related to the menstrual cycle, 31 for a problem relating to the menopause, 11 for a combination of these and 12 for another reason (for example, pre-conception advice); 171 were granted funding and seen in the clinic, 146 for a problem relating to the menstrual cycle, providing the sample for analysis.

The 146 patients seen in clinic for a menstrual problem had a mean age of 35 years (s.d. = 9.3, range 13–55). A psychiatric diagnosis was made in 130 patients (89.0%); a minority of 16 (11.0%) had no diagnosis. The primary psychiatric diagnosis was depressive disorder in 46 (31.5%), bipolar affective disorder in 20 (13.7%), anxiety disorder in 19 (13.0%), neurodevelopmental disorder in 15 (10.2%), personality disorder in 12 (8.2%), schizophrenia spectrum disorder in 5 (3.4%) and other psychiatric disorders in 4 (2.7%); 50 patients had more than one psychiatric diagnosis, the most common secondary diagnosis being anxiety disorder, present in 18 (12.3%).

At the time of assessment, 65 (44.5%) were prescribed antidepressant medication, 29 (19.9%) were prescribed antipsychotics, 16 (11.0%) were prescribed mood stabilisers and 36 (24.6%) were prescribed other psychiatric medications. A minority of 51 (34.9%) were not prescribed psychiatric medication at the time of assessment.

Following assessment, 94 (64.4%) met criteria for probable PMDD and 67 (45.9%) for probable exacerbation of an underlying psychiatric disorder. In 34 (24.0%), it was diagnostically uncertain whether the patient was experiencing PMDD, exacerbation of an underlying disorder or a combination of both. Notably, only 33 (22.6%) provided prospective menstrual diaries, which is recommended for definitive diagnosis of premenstrual disorders.^4^ The primary psychiatric diagnoses of those experiencing PMDD and premenstrual exacerbation are shown in Table 1.

**Table 1:** Participants classified as having premenstrual dysphoric disorder or premenstrual exacerbation of an underlying psychiatric disorder following assessment, divided by primary psychiatric diagnosis (*n* = 146)

Primary psychiatric diagnosis	Premenstrual dysphoric disorder, *n*		Premenstrual exacerbation, *n*	
	Yes	No	Yes	No
Anxiety disorders	11	8	11	8
Bipolar disorder	11	9	13	7
Depression	31	15	26	20
Neurodevelopmental disorders	8	7	9	6
None	22	3	0	25
Other	1	3	0	4
Personality disorders	9	3	5	7
Schizophrenia spectrum disorders	1	4	3	2
Total	94	52	67	79

Depressive symptoms were most associated with the menstrual cycle, experienced by 130 (89.0%) of patients; 15 (10.2%) reported menstrual migraines, 7 (4.8%) had manic or hypomanic symptoms associated with their cycle and 3 (2.1%) had associated psychotic symptoms. Of the 13 patients experiencing premenstrual exacerbation of bipolar affective disorder, all experienced cyclical worsening of depressive symptoms and 2 also had exacerbation of hypomanic/manic symptoms.

There was at least one comorbid gynaecological condition in 68 patients (46.6%): 20 (13.7%) had polycystic ovary syndrome, 10 (6.9%) endometriosis, 48 (32.9%) menorrhagia or dysmenorrhoea and 16 (14.4%) other gynaecological problems. At the time of assessment, 37 (25.5%) were prescribed hormonal contraception or medication for a gynaecological problem: 17 (11.4%) oral contraception, 10 (6.9%) transdermal oestrogen, 1 (0.7%) a gonadotrophin-releasing hormone analogue, 8 (5.5%) a progestogen-emitting intrauterine system, 3 (2.1%) a copper-emitting intrauterine device and 5 (3.4%) hormone replacement therapy.

## Discussion

This study describes the clinical characteristics of 146 consecutive referrals for premenstrual disorders to the UK's National Female Hormone Clinic over a 6-year period. Most of the patients attending the clinic had a comorbid psychiatric disorder; a minority of the sample had no psychiatric diagnosis or were not prescribed psychotropic medication.

Almost half the sample were found to have premenstrual exacerbation of an underlying psychiatric disorder, encompassing a range of diagnoses. Depressive symptoms were most commonly associated with the menstrual cycle. It is widely recognised that both unipolar^1^ and bipolar^5^ affective disorders can be exacerbated premenstrually. Of those experiencing premenstrual exacerbation of bipolar disorder, all had exacerbation of depressive symptoms. This is in keeping with one study suggesting that the menstrual cycle can trigger depressive but not manic episodes^6^ but is in contrast to a systematic review's finding that case reports of mania or hypomania exacerbated by the cycle were more common than reports of exacerbated depression.^5^

Only five patients attending the clinic had a diagnosis of a schizophrenia spectrum disorder, three of whom experienced premenstrual exacerbation. This low number is in contrast to a recent meta-analysis demonstrating a clear excess of admissions for psychosis during the perimenstrual phase,^7^ which may relate to the relative difficulty that patients with psychotic disorders face accessing the clinic.

Since anxiety is a diagnostic feature of PMDD, it is unsurprising that anxiety disorders showed premenstrual exacerbation. Neurodevelopmental disorders have been less researched in relation to the menstrual cycle but emerging evidence suggests that autism^8^ and attention-deficit hyperactivity disorder^9^ exhibit exacerbation of symptoms. There has also been a small but well-conducted prospective study to suggest a substantial proportion of females with borderline personality disorder demonstrate premenstrual exacerbation.^10^

The difficulty in distinguishing PMDD from premenstrual exacerbation is highlighted by a quarter of our sample being categorised with both conditions. Although there are likely to be some who experience both these conditions concurrently, the diagnostic challenge in differentiating them is well-known.^2^ The use of prospective menstrual diaries may improve classification, but these were only available for a small proportion of our sample at baseline assessment. In six cases, the treatment recommendation included prospectively recording symptoms in relation to the menstrual cycle to provide diagnostic clarity. Future service development will include systematically requesting that patients complete menstrual cycle symptom diaries prior to attending the clinic.

### Limitations

Our study has some important limitations. First, as it is derived from patients attending a national specialist service, the sample is not representative of the wider population who experience premenstrual disorders. Second, diagnoses were based on clinical assessment rather than structured clinical interview or prospective diaries, although the assessments were done by a single senior consultant psychiatrist who also had training in gynaecology. Third, as a specialist advisory service, the clinic provides no systematic follow-up of patients and therefore this study is cross-sectional in nature.

### Implications

Our findings suggest that expertise in both psychiatry and gynaecology is necessary to manage premenstrual disorders in individuals referred to specialist services.

## Data Availability

Data not available due to ethical restrictions. As this was an audit, patient consent for data sharing was not given.

## About the authors

**Thomas J. Reilly** is an honorary specialist registrar in general adult psychiatry in the National Female Hormone Clinic at South London and Maudsley NHS Foundation Trust and MRC Clinical Training Research Fellow in the Department of Psychosis Studies at the Institute of Psychiatry, Psychology and Neuroscience, King's College London, UK. **Clare-Louise Knox** is a PhD candidate in the Department of Psychological Medicine at the Institute of Psychiatry, Psychology and Neuroscience, King's College London, UK. **Michael S. Marsh** is a consultant gynaecologist and obstetrician in the Department of Obstetrics and Gynaecology at King's College Hospital NHS Foundation Trust and the National Female Hormone Clinic, South London and Maudsley NHS Foundation Trust, an honorary senior lecturer at King's College School of Medicine and an honorary senior lecturer at the Institute of Psychiatry, Psychology and Neuroscience, King's College London, UK. **Michael C. Craig** is a consultant psychiatrist in the National Female Hormone Clinic, South London and Maudsley NHS Foundation Trust and Professor of Translational Reproductive and Neurodevelopmental Sciences at the Institute of Psychiatry, Psychology and Neuroscience, King's College London, UK.
